# Influence of Ethnicity and Deprivation on Occurrence of Paget’S Disease in Greater Manchester, UK

**DOI:** 10.1007/s00223-024-01297-y

**Published:** 2024-10-23

**Authors:** A. H. Heald, W. Lu, R. Williams, K. McCay, A. Maharani, M. J. Cook, T. W. O’Neill

**Affiliations:** 1 The School of Medicine and Manchester Academic Health Sciences Centre, Manchester University, Manchester, UK; 2 Department of Endocrinology and Diabetes, Salford Royal Hospital, Salford, M6 8HD UK; 3 Department of Computing & Mathematics, Faculty of Science and Engineering, Manchester Metropolitan University, Manchester, UK; 4 Division of Informatics, Imaging and Data Science, Faculty of Biology, Medicine and Health, University of Manchester, Manchester, UK; 5 NIHR Applied Research Collaboration Greater Manchester, Manchester Academic Health Science Centre, University of Manchester, Manchester, UK; 6 Division of Nursing, Midwifery and Social Work, School of Health Sciences, Faculty of Biology, Medicine, and Health, The University of Manchester and Manchester Academic Health Science Centre (MAHSC), Manchester, UK; 7 Centre for Epidemiology Versus Arthritis, Division of Musculoskeletal and Dermatological Sciences, Faculty of Biology Medicine and Health, University of Manchester, Manchester, UK; 8 NIHR Manchester Biomedical Research Centre, Manchester University NHS Foundation Trust, Manchester Academic Health Science Centre, Manchester, UK; 9 Department of Rheumatology, Northern Care Alliance, Manchester, UK

**Keywords:** Paget’s disease, Epidemiology, Ethnicity, Deprivation, COVID-19

## Abstract

There is important variation in the occurrence of Paget’s disease in different regions and populations. There are though few data concerning the occurrence of clinically diagnosed disease in black and ethnic minority groups in the United Kingdom (UK). We undertook an anonymised search using an integrated primary and secondary care-based database in Greater Manchester, covering a population of over 3 million people. We looked also among those with a first positive COVID test, the influence of Paget’s disease on subsequent admission to hospital within 28 days. Within our database, there were 534,571 people aged 60 years and over alive on 1 January 2020. The majority were white (84%) with 4.7% identifying as Asian or Asian British, and 1.27% Black or Black British. There were 931 with clinically diagnosed Paget’s disease. Overall prevalence in the greater Manchester area was 0.174%. Prevalence was higher in men than women (0.195 vs 0.155%). Compared to the prevalence of Paget’s in whites (0.179%) the prevalence was lower among those identifying as Asian or Asian British (0.048%) and higher among those identifying as Black or Black British (0.344%). Prevalence increased with increasing deprivation. Clinically diagnosed Paget’s disease is uncommon affecting 0.174% of men and women aged 60 or more years. Within Greater Manchester, it was more common in those identifying as Black or Black British and less common in those identifying as Asian or Asian British.

## Introduction

There is evidence of variation in occurrence of Paget’s disease worldwide with the disease most frequent in the UK; and evidence also of a decline in frequency and severity of the disease in different populations over the past 50 years [1–3]. Data from recent population studies suggests a decline also in the incidence of clinically diagnosed disease in the UK though there are no recent UK data concerning the *prevalence* of clinically diagnosed disease [4, 5]. Paget’s disease has been traditionally considered to be uncommon in Asia and rural Africa [6–10]. Data from Johannesburg in South Africa and USA, however, suggest a prevalence of radiographic disease of over 1% among Blacks, and that the frequency in some areas is comparable with the prevalence in whites [11–13]. There are clinical reports of Paget’s disease in South Asians from the Indian subcontinent and Blacks in the UK [14]. However, to date there are no population data concerning the prevalence of clinically diagnosed disease in these groups. There is some evidence that levels of deprivation may impact on the occurrence of disease, and it is important therefore to consider whether any observed variation in disease frequency in different population groups could be explained by variation in socioeconomic status [5].

Using data from a large primary and secondary linked register within Greater Manchester the aim of this study was to determine the occurrence of clinically diagnosed Paget’s disease and the influence of age, ethnicity and socioeconomic status on occurrence. We looked also at the influence of Paget’s disease on the likelihood of admission to hospital following a first COVID-19 positive test.

### Methods

#### Subjects

We undertook an anonymised search using the Greater Manchester Care Record (GMCR) database. The GMCR is an integrated database of primary care, secondary care and mental health trusts from across Greater Manchester (https://gmwearebettertogether.com/research-and-planning/: accessed 18 August 2023) for retrospective analyses covering a population of approximately 3 million people. Health and care data were collected from 433 of 435 (99.5%) general practices in Greater Manchester. The 2 GP surgeries that do not contribute data have chosen to opt out of data sharing into the GMCR. For reference, one is located in Tameside and the other is in Bolton. Data were de-identified at source and were extracted from the GMCR database. Coded diagnoses were according to the READ code system historically (https://www.scimp.scot.nhs.uk/better-information/clinical-coding/scimp-guide-to-read-codes: accessed 20 July 2023) and more recently the SNOMED classification (SNOMED CT—NHS Digital: accessed 20 July 2023). We reviewed the health records of anyone aged 60 or over living in Greater Manchester on 1st January 2020.

#### Ethics

This project was reviewed and ethically approved by Health Innovation Manchester and granted by the Greater Manchester Care Record (GMCR) review board (ref: IDCR-RQ-038). This research was performed with anonymised data, in line with the Health Research Authority’s Governance arrangements for research ethics committees.

#### Variables

We identified those individuals in the data set who had a code for Paget’s disease (see Box). Deprivation was assessed using the Townsend score [15]. The Townsend score is based on UK postcode and can be calculated using a combination of four census variables for any geographical area (provided census data is available for that area). The measure has been widely used in research on health, education and crime to establish whether relationships exist with deprivation. A higher Townsend score equates to greater social disadvantage. Information was provided by quintile using categorisations based on published data from the UK (https://statistics.ukdataservice.ac.uk/dataset/2011-uk-townsend-deprivation-scores accessed 29 Dec 2023). Ethnic group was assigned by Graphnet prior to data extraction, using an algorithm drawing on multiple electronic health record sources for each individual. NHS ethnic group categories were recoded according NHS 5 groups (https://datadictionary.nhs.uk/data_elements/ethnic_category.html: Web accessed 14 August 2023). During the pandemic information about the date of people’s COVID-19 positive tests was recorded centrally and linked to the GMCR. Information concerning hospital admissions and the date of those admissions was also included. We defined severe COVID-19 as those who had a positive test and were admitted to hospital anywhere between 4 days before and 28 days after a positive test.

Box. Codes used to identify people with Paget’s disease of bone.

**Table:** 

Terminology	Clinical code	Description
ctv3	BBV5	Osteosarcoma in Paget's disease of bone
ctv3	N31.	Osteitis deformans and osteopathies associated with diseases EC
ctv3	N310	Osteitis deformans
ctv3	N310	Paget's disease of bone
ctv3	N3100	Paget's disease-cervical spine
ctv3	N3101	Paget's disease-thoracic spine
ctv3	N3102	Paget's disease-lumbar spine
ctv3	N3103	Paget's disease-sacrum
ctv3	N3105	Paget's disease-clavicle
ctv3	N3106	Paget's disease-scapula
ctv3	N3107	Paget's disease of humerus
ctv3	N3108	Paget's disease-radius
ctv3	N3109	Paget's disease-ulna
ctv3	N310A	Paget's disease-carpal bone
ctv3	N310D	Paget's disease of pelvis
ctv3	N310E	Paget's disease-femur
ctv3	N310F	Paget's disease-patella
ctv3	N310G	Paget's disease-tibia
ctv3	N310H	Paget's disease-fibula
ctv3	N310L	Paget's disease-other tarsal bone
ctv3	N310P	Paget's disease of skull
ctv3	N310x	Paget's disease-multiple sites
ctv3	N310y	Paget's disease OS
ctv3	N310z	Paget's disease NOS
ctv3	N311	Osteitis deformans associated with diseases EC
ctv3	N3110	Osteitis deformans in neoplastic disease
ctv3	NyuCD	[X]Osteitis deformans in neoplastic diseases classified elsewhere
ctv3	N3104	Paget's disease-coccyx
ctv3	N310B	Paget's disease-metacarpal
ctv3	N310C	Paget's disease-phalanx of finger or thumb
ctv3	N310J	Paget's disease-calcaneum
ctv3	N310K	Paget's disease-talus
ctv3	N310M	Paget's disease-metatarsal
ctv3	N310N	Paget's disease-phalanx of toe
ctv3	X20Sp	Paget's disease of jaw
ctv3	Xa7ns	Pagets disease—hip
emis	^ESCTOS253195	Osteitis deformans
emis	^ESCTOS304203	Osteosarcoma in Paget's disease of bone
emis	^ESCTOS304204	Osteosarcoma in Paget disease of bone
emis	^ESCTOS481980	Osteitis deformans and osteopathies associated with other diseases
emis	^ESCTOS481981	Osteitis deformans and osteopathies associated with diseases EC
emis	^ESCTOS482029	Osteitis deformans of skull
emis	^ESCTOS482032	Osteitis deformans associated with another disorder
emis	^ESCTPA253197	Pagets disease of bone
emis	^ESCTPA481983	Paget disease-cervical spine
emis	^ESCTPA481985	Paget disease-thoracic spine
emis	^ESCTPA481987	Paget disease-lumbar spine
emis	^ESCTPA481989	Paget disease-sacrum
emis	^ESCTPA481993	Paget disease-clavicle
emis	^ESCTPA481995	Paget disease-scapula
emis	^ESCTPA481996	Paget's disease of humerus
emis	^ESCTPA481997	Paget disease of humerus
emis	^ESCTPA481999	Paget disease-radius
emis	^ESCTPA482001	Paget disease-ulna
emis	^ESCTPA482003	Paget disease-carpal bone
emis	^ESCTPA482009	Paget disease of pelvis
emis	^ESCTPA482012	Paget disease-femur
emis	^ESCTPA482014	Paget disease-patella
emis	^ESCTPA482016	Paget disease-tibia
emis	^ESCTPA482018	Paget disease-fibula
emis	^ESCTPA482027	Paget's disease of skull
emis	^ESCTPA482028	Paget disease of skull
emis	^ESCTPA482031	Paget disease-multiple sites
emis	^ESCTOS419307	Osteitis deformans without bone tumour
emis	^ESCTOS419308	Osteitis deformans without bone tumour
emis	^ESCTOS507540	Osteitis deformans of jaw
emis	^ESCTOS750378	Osteoporosis circumscripta
emis	^ESCTPA481991	Paget disease-coccyx
emis	^ESCTPA482005	Paget disease-metacarpal
emis	^ESCTPA482007	Paget disease-phalanx of finger or thumb
emis	^ESCTPA482020	Paget disease-calcaneum
emis	^ESCTPA482022	Paget disease-talus
emis	^ESCTPA482024	Paget disease-metatarsal
emis	^ESCTPA482026	Paget disease-phalanx of toe
emis	^ESCTPA507539	Paget's disease of jaw
emis	^ESCTPA507541	Paget disease of jaw
emis	^ESCTPA588812	Pagets disease—hip
emis	^ESCTSP788085	Spastic paraplegia with Paget disease of bone syndrome
readv2	N310.11	Paget's disease of bone
readv2	N310D00	Paget's disease-pelvis
readv2	N310G00	Paget's disease-tibia
readv2	N310z00	Paget's disease NOS
readv2	N310.00	Osteitis deformans—Paget's disease of the bone
readv2	N310000	Paget's disease-cervical spine
readv2	N310P00	Paget's disease-skull
readv2	N31..00	Osteitis deformans/osteopathies associated with diseases EC
readv2	N310200	Paget's disease-lumbar spine
readv2	N310y00	Paget's disease OS
readv2	N311.00	Osteitis deformans associated with diseases EC
readv2	N310300	Paget's disease-sacrum
readv2	N310F00	Paget's disease-patella
readv2	N310E00	Paget's disease-femur
readv2	BBV5.00	[M]Osteosarcoma in Paget's disease of bone
readv2	N310700	Paget's disease-humerus
readv2	N310800	Paget's disease-radius
readv2	N310 × 00	Paget's disease-multiple sites
readv2	N310500	Paget's disease-clavicle
readv2	N310600	Paget's disease-scapula
readv2	N310100	Paget's disease-thoracic spine
readv2	N310H00	Paget's disease-fibula
readv2	N310900	Paget's disease-ulna
readv2	N310A00	Paget's disease-carpal bone
readv2	N311000	Osteitis deformans in neoplastic disease
readv2	N310L00	Paget's disease-other tarsal bone
readv2	NyuCD00	[X]Osteitis deformans in neoplastic diseases classified elsewhere
readv2	N310400	Paget's disease-coccyx
readv2	N310B00	Paget's disease-metacarpal
readv2	N310C00	Paget's disease-phalanx of finger or thumb
readv2	N310J00	Paget's disease-calcaneum
readv2	N310K00	Paget's disease-talus
readv2	N310M00	Paget's disease-metatarsal
readv2	N310N00	Paget's disease-phalanx of toe
snomed	203,326,004	Osteitis deformans and osteopathies associated with diseases EC (disorder)
snomed	203,327,008	Paget's disease-cervical spine (disorder)
snomed	203,328,003	Paget's disease-thoracic spine (disorder)
snomed	203,329,006	Paget's disease-lumbar spine (disorder)
snomed	203,330,001	Paget's disease-sacrum (disorder)
snomed	203,332,009	Paget's disease-clavicle (disorder)
snomed	203,333,004	Paget's disease-scapula (disorder)
snomed	203,334,005	Paget's disease of humerus (disorder)
snomed	203,335,006	Paget's disease-radius (disorder)
snomed	203,336,007	Paget's disease-ulna (disorder)
snomed	203,337,003	Paget's disease-carpal bone (disorder)
snomed	203,340,003	Paget's disease of pelvis (disorder)
snomed	203,342,006	Paget's disease-femur (disorder)
snomed	203,343,001	Paget's disease-patella (disorder)
snomed	203,344,007	Paget's disease-tibia (disorder)
snomed	203,345,008	Paget's disease-fibula (disorder)
snomed	203,351,003	Paget's disease of skull (disorder)
snomed	203,352,005	Paget's disease-multiple sites (disorder)
snomed	203,355,007	Osteitis deformans associated with diseases EC (disorder)
snomed	203,356,008	Osteitis deformans in neoplastic disease (disorder)
snomed	2,089,002	Osteitis deformans (disorder)
snomed	33,681,003	Osteosarcoma in Paget's disease of bone (morphologic abnormality)
snomed	314,961,000,119,103	Paget disease of multiple vertebra (disorder)
snomed	726,622,002	Spastic paraplegia with Paget disease of bone syndrome (disorder)
snomed	235,117,006	Paget's disease of jaw (disorder)
snomed	698,047,001	Osteoporosis circumscripta (disorder)
snomed	203,350,002	Paget's disease-phalanx of toe (disorder)
snomed	203,349,002	Paget's disease-metatarsal (disorder)
snomed	203,347,000	Paget's disease-talus (disorder)
snomed	203,346,009	Paget's disease-calcaneum (disorder)
snomed	314,941,000,119,102	Paget disease of right femur (disorder)
snomed	315,051,000,119,100	Paget disease of left femur (disorder)
snomed	301,027,009	Pagets disease—hip (disorder)
snomed	203,331,002	Paget's disease-coccyx (disorder)
snomed	203,339,000	Paget's disease-phalanx of finger or thumb (disorder)
snomed	203,338,008	Paget's disease-metacarpal (disorder)
snomed	1,077,851,000,119,108	Paget disease of right scapula (disorder)
snomed	1,077,861,000,119,105	Paget disease of left scapula (disorder)
snomed	111,254,007	Osteitis deformans without bone tumour (disorder)

#### Statistics

Descriptive statistics were used to characterise the population, including the number of men and women in different age categories (60–64 yrs; 65–69 yrs; 70–74 yrs; 75–79 yrs; 80–84 yrs and 85 yrs and over) and number in each of the Townsend quintiles and also ethnic groups. We looked at the occurrence of Paget’s disease in each of these groups.

We used logistic regression to explore the association between Paget’s disease (outcome) and predictor variables, including age (expressed as a continuous variable), ethnicity (using whites as the reference group), gender (using females as the reference group) and Townsend quintile (using the most affluent first quintile as reference) with the results expressed as odds ratios (OR) and 95% confidence intervals (CI). We looked initially at the association between Paget’s disease and each of the predictor variables unadjusted for any other covariates (model 1), and subsequently after adjustment for age and gender (model 2) and after adjustment for all covariates (model 3).

We looked then among those people who had a first positive COVID test, the influence of Paget’s disease on whether they were admitted to hospital within 28 days with adjustments made for age, gender, Townsend quintile and ethnicity. The exact numbers in each analysis differed slightly in relation to the specific analysis conducted.

## Results

### Descriptive Statistics

There were 534,571 people alive on 1 January 2020 who were 60 years of age or older in Greater Manchester. Of these, 254,125 were men (47.5%) with a mean age of 72 yrs (SD 8 yrs) and 280,442 (52.5%) women with a mean age of 73 yrs (SD = 9 yrs). The numbers of men and women by age band are shown in Table 1. Using national quintiles of the Townsend Index (see Table 1) there was a slightly higher than expected proportion of people in both the least deprived (first) quintile (23.50%) and also in the most deprived (fifth) quintile (21.70%) with slightly lower than expected proportions of people in the intermediate (2nd, 3rd and 4th) quintiles. Regarding ethnicity, ethnic white individuals made up 84.03% of the population of people 60 years old or more, with 4.72% Asian or Asian British, 1.27% Black or Black British and 0.54% reported as of mixed ethnic group. ‘Other’ ethnic groups made up 3.26% of the population with 6.18% not wishing to declare ethnicity, see Table 1.

**Table 1:** Subject characteristics

	N (%)	Men	Women
*Age Group in years*			
60–64 yrs	133,021 (24.88%)	68,056	64,965
65–69 yrs	113,050 (21.15%)	56,496	56,552
70–74 yrs	109,009 (20.39%)	52,751	56,256
75–79 yrs	77,450 (14.49%)	35,858	41,592
80–84 yrs	54,803 (10.25%)	24,016	30,787
> = 85 yrs	47,238 (8.84%)	16,948	30,290
Townsend quintile *			
1	125,566 (23.50%)	59,745	65,821
2	97,250 (18.20%)	45,567	51,682
3	93,140 (17.43%)	43,616	49,523
4	102,417 (19.17%)	48,559	53,858
5	115,965 (21.70%)	56,506	59,457
*Ethnic Group*			
Asian or Asian British	24,757 (4.72%)	12,310	12,447
Black or Black British	6,688 (1.27%)	3,255	3,433
Mixed	2,850 (0.54%)	1,400	1,450
Other Ethnic Groups	17,083 (3.26%)	8,324	8,758
Refused and not stated group	32,464 (6.18%)	16,101	16,363
White	441,071 (84.03%)	207,221	233,847

^*^Townsend quintiles: 1 (< − 3.308); 2 (− 3.308, − 1.6918); 3 (− 1.6918, 0.4866); 4 (0.4866, 3.2828); 5 (> = 3.2828)

### Prevalence of Paget’S Disease

**Fig. 1:**
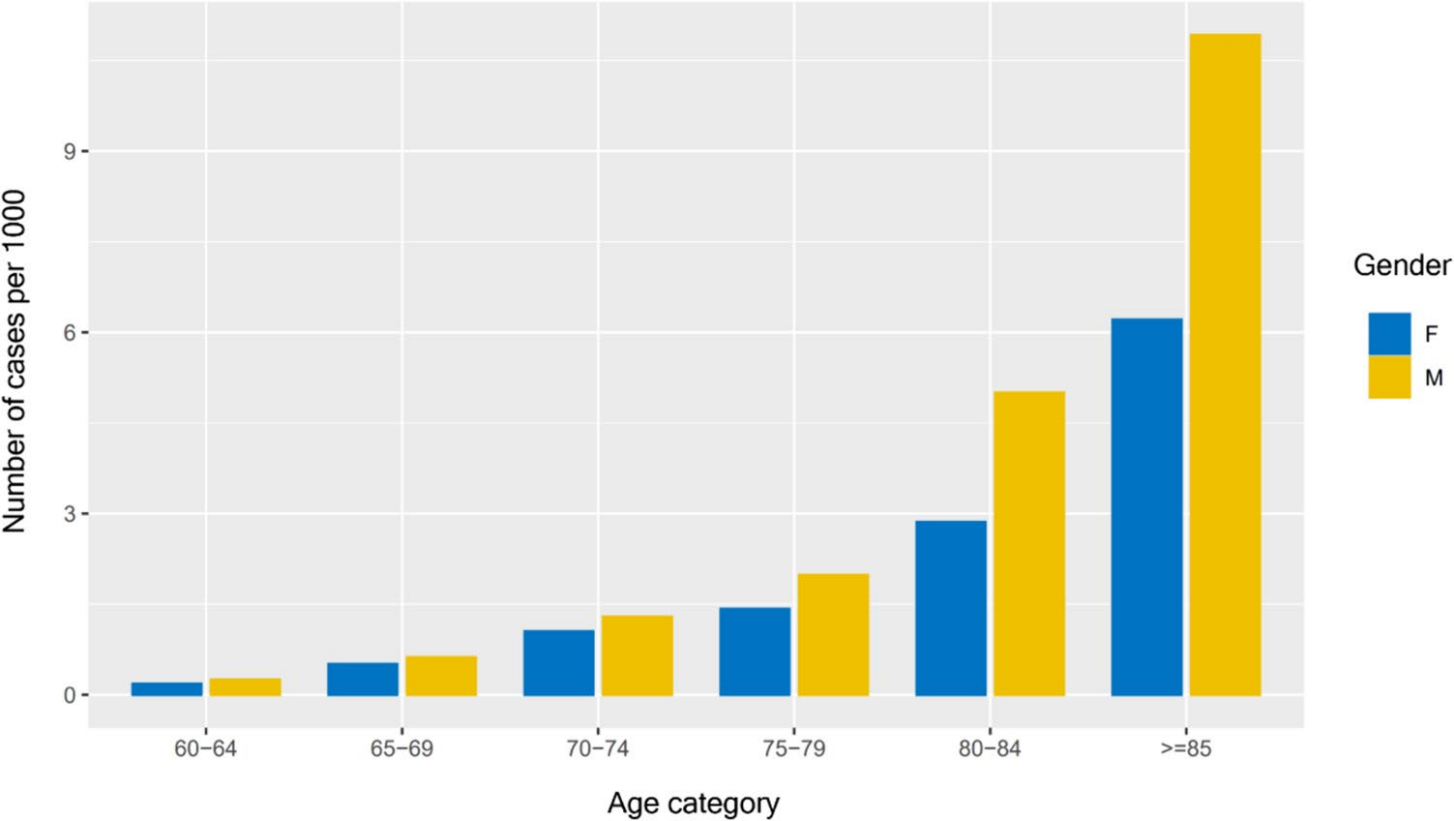
Prevalence of Paget’s disease – Influence of Age and Gender

931 (0.174%) men and women had a diagnostic code for Paget’s disease in the clinical record, see Table 2. The prevalence was, as expected, greater in men than women (0.195% vs 0.155%) and increased with age, from 0.022% at age 60–64 yrs rising to 0.79% at age 85 yrs and over, see Table 2 and Fig. 1. Prevalence increased with increasing quintile of Townsend score from the least deprived area (0.164%) to the second most deprived area (0.189%), with a fall among those in the most deprived quintile (0.173%), with a similar pattern in men and women. Paget’s was most frequent among identifying as Black or Black British (0.344%) and least frequent among those identifying as Asian or Asian British (0.048%). The prevalence of Paget’s among whites was 0.179%.

**Table 2:** Prevalence of Paget’s Disease: By age, gender, ethnicity and deprivation level

	All		Men		Women	
	N	Prevalence %	N	%	N	%
All	931	0.174%	496	0.195%	435	0.155%
*Age Group*						
60–64 yrs	29	0.022%	17	0.025%	12	0.018%
65–69 yrs	64	0.057%	35	0.062%	29	0.051%
70–74 yrs	127	0.117%	68	0.129%	59	0.105%
75–79 yrs	130	0.168%	71	0.198%	59	0.142%
80–84 yrs	208	0.380%	120	0.500%	88	0.286%
> = 85 yrs	373	0.790%	185	1.092%	188	0.621%
*Townsend quintile (score)*						
1	206	0.164%	122	0.204%	84	0.128%
2	164	0.169%	87	0.191%	77	0.149%
3	166	0.178%	84	0.193%	82	0.166%
4	194	0.189%	101	0.208%	93	0.173%
5	201	0.173%	102	0.181%	99	0.167%
*Ethnic Group*						
Asian or Asian British	12	0.048%	5	0.041%	7	0.056%
Black or Black British	23	0.344%	13	0.399%	10	0.291%
Mixed	6	0.211%	3	0.214%	3	0.207%
Other Ethnic Groups	28	0.164%	14	0.168%	14	0.160%
Refused and not stated group	65	0.200%	27	0.168%	38	0.232%
White	791	0.179%	431	0.208%	360	0.154%

^*^Townsend quintiles: 1 (< − 3.308); 2 (− 3.308, − 1.6918); 3 (− 1.6918, 0.4866); 4 (0.4866, 3.2828); 5 (> = 3.2828)

### Regression Analysis

In an unadjusted logistic regression analysis Paget’s was associated with increasing age (OR = 1.12; 95% CI 1.11, 1.13), and male gender (OR (vs female) = 1.26; 95% CI 1.11, 1.43), see Table 3. Compared to whites there was an increased risk among those identifying as Black or Black British (OR = 1.92; 95% CI 1.23, 2.83) and a reduced risk among those identifying as Asian or Asian British (OR (vs white) = 0.27; 95% CI 0.14, 0.46). There was a small increase in risk linked with increasing Townsend quintile to the second most deprived quintile though the confidence intervals embraced unity. After adjustment for initially age and gender (model 2) and after mutual adjustment for all variables (model 3) the magnitude of the association with age was similar (OR = 1.12). After mutual adjustment (model 3) the strength of the association with male gender increased (OR = 1.65) and there was a gradual increase in risk with increasing Townsend score across all quintiles with evidence of a significant trend. Among those identifying as Asian or Asian British the strength of the association was attenuated (OR = 0.36) while among those identifying as Black or Black British the strength of the association was more marked (OR = 2.13), see Table 3.

**Table 3:** Influence of age, gender, ethnicity and deprivation on occurrence of Paget’s disease

	Model 1	Model 2	Model 3
	Odds Ratio (95% CI)	Odds Ratio (95% CI)	Odds Ratio (95% CI)
*Sex*			
Women (Referent)	1	1	1
Men	1.26 (1.11, 1.43)	1.63 (1.44, 1.86)	1.65 (1.45, 1.88)
Age	1.12 (1.11, 1.13)	1.12 (1.11, 1.13)	1.12 (1.12, 1.13)
*Townsend (quintile)*			
1 Highest (Referent)	1	1	1
2	1.03 (0.84, 1.26)	1.00 (0.81, 1.22)	1.00 (0.81, 1.22)
3	1.09 (0.88, 1.33)	1.04 (0.85, 1.28)	1.04 (0.85, 1.28)
4	1.15 (0.95, 1.41)	1.11 (0.91, 1.35)	1.11 (0.91, 1.35)
5 Lower	1.06 (0.87, 1.28)	1.14 (0.93, 1.38)	1.15 (0.95, 1.41)
*Ethnic Group*			
White (Referent)	1	1	1
Asian or Asian British	0.27 (0.14, 0.46)	0.38 (0.20, 0.64)	0.36 (0.19, 0.62)
Black or Black British	1.92 (1.23, 2.83)	2.26 (1.45, 3.34)	2.13 (1.35, 3.17)
Mixed	1.17 (0.47, 2.39)	1.42 (0.56, 2.89)	1.38 (0.55, 2.82)
Other	0.91 (0.61, 1.31)	0.98 (0.66, 1.40)	0.97 (0.65, 1.39)
Refused	1.12 (0.86, 1.43)	0.83 (0.63, 1.06)	0.83 (0.63, 1.06)

^*^Townsend quintiles: 1 (< − 3.308); 2 (− 3.308, − 1.6918); 3 (− 1.6918, 0.4866); 4 (0.4866, 3.2828); 5 (> = 3.2828)

Model 1 – unadjusted; Model 2, adjusted for age and gender; Model 3, mutually adjusted

### Risk of Hospital Admission Following a First Positive COVID Test

Within the cohort there were 86,844 people who had a positive COVID test recorded in their clinical record. Of these 11% had a hospital admission up to + 28 days or up to 4 days prior to the test. After mutual adjustment (age, gender, ethnicity, Townsend quintile), as expected the risk of admission increased with increasing age (OR per year = 1.05; 95% CI 1.05, 1.06), was greater in men than women (OR 1.38; 95% CI 1.32, 1.44), increased with Townsend quintile (OR most deprived vs least deprived = 1.98; 95% CI 1.85, 2.11) and was more common in those identifying as Asian of Asian British (OR = 1.55; 95% CI 1.42, 1.70) and Black or Black British (OR1.83; 95% CI 1.54, 2.16), see Table 4. Among those with a positive COVID-19 test those with Paget's were more likely to require admission to hospital within 28 days, (OR 1.37; 95% CI (0.94, 1.95). Thus there was a 37% increased risk of admission among those with Paget’s disease. However the confidence interval embraced unity.

**Table 4:** Influence of Paget’s disease on likelihood of hospitalisation within 28 days of 1st COVID positive test

	Odds ratio (95% CI)
Paget diagnosis (yes vs no)	1.37 (0.94, 1.95)
Age (years)	1.05 (1.05, 1.06)
Gender (men vs women)	1.38 (1.32, 1.44)
Ethnicity White Asian or Asian British Black or Black British Mixed OtherRefused	1.00 1.55 (1.42, 1.70) 1.83 (1.54, 2.16) 1.15 (0.82, 1.58) 1.03 (0.90, 1.18) 1.37 (1.25, 1.49)
Townsend (Quintiles) 1 2 3 4 5	1.00 .16 (1.08, 1.25) 1.31 (1.22, 1.41) 1.48 (1.38, 1.58) 1.98 (1.85, 2.11)

## Discussion

In this population-based study of men and women greater than 60 years residing in Greater Manchester, UK the prevalence of clinically diagnosed Paget’s disease was 0.174%. Compared to those who were white, prevalence was greater among those identifying as Black or Black British (0.344%) and lower among those identifying as Asian or Asian British (0.048%). There was a small increase in the likelihood of disease with increasing levels of deprivation. After adjustment for other factors linked with poor COVID outcomes, those with Paget’s disease had a small though non-significant increase in the risk of admission to hospital within 28 days of a positive COVID test.

Our data are consistent with previous studies showing an increase in occurrence of clinically diagnosed Paget’s disease with increasing age and a greater incidence in men than women [4]. Our findings are also consistent with data from the UK general practice research database suggesting an increase in risk with increasing deprivation [5]. Also recent data from Quebec, Canada, using data from health administrative databases, suggesting a link with increasing social and material deprivation [16]. The explanation for this remains uncertain; selection bias seems unlikely as those living in areas of greater deprivation are, if anything, less likely to consult their primary care physician and thus to be clinically diagnosed. Supporting the view that socioeconomic factors may influence occurrence is the observation in a recent case–control study of a link between Paget’s disease and low education level[17].

Recent studies have reported a decline in the incidence of clinically diagnosed Paget’s over the past 30 years [4, 5]. Based on data obtained during 1988–1999 it was estimated (using incidence and mortality rates) that the prevalence of clinically diagnosed disease among those age 55 years and older was 0.3%. Our data provide a robust estimate of the current prevalence and suggest that 0.174% of people aged 60 years and over have clinically diagnosed disease.

How do our data compare with findings relating to the occurrence of radiographic disease. The most recent data concerning radiographic prevalence derives from a survey of 1,000 stored abdominal and pelvic CT images in Lancaster, UK [18]. Evaluation of the images suggests a radiographic prevalence of 0.8% in men and women aged 55 years and over. Comparison with our findings suggests that somewhere between one in four and one in five of those with radiographic evidence of the disease will come to clinical attention. Caution however is needed in extrapolating these data to other parts of the UK as there is important geographic variation in disease occurrence with rates highest in the NW England [1, 2].

In our study we found a higher prevalence of clinically diagnosed disease among blacks than whites. To our knowledge there are no previous data relating to the population occurrence of Paget’s disease in black people in the UK. Evidence from sub-Saharan Africa suggest a relatively low prevalence in native Africans. However, in a radiological survey in Johannesburg, South Africa Guyer reported a radiographic prevalence of 1.3% in blacks compared with a prevalence of 2.4% among whites [12]. In a survey of two cities in USA (New York and Atlanta) the prevalence of disease was found to be slightly higher in whites than blacks in New York (3.9% vs 2.6%) [11]. In Atlanta, however, interestinglyPaget’s Disease was slightly greater among blacks than whites in Atlanta (1.2% vs 0.9%), and among Atlanta men the disease was twice as frequent among blacks (1.9% vs 0.9%). In a more recent analysis of patients attending the Birmingham (USA) VA Medical Centre over a 20 year period, Paget’s disease appeared to be more common among African American than White patients (0.51% vs 0.4%) [19]. Using data from NHANES 1 the prevalence of Paget’s (based on information from pelvic radiographs) was similar in whites and blacks (0.72% vs 0.73%) [13].

Asians living in the greater Manchester area are of predominantly Indian, Pakistani and Bangladeshi origin. To our knowledge there are no radiographic survey data concerning occurrence of Paget’s in the Indian subcontinent or data concerning occurrence of Paget’s among Asians who live elsewhere. There were no cases of Paget’s reported among those of Asian background in an NHANES survey using pelvic radiographs, however, the numbers studied were small (*n* < 38) and their Asian origin was not specified [13]. Although traditionally considered to be uncommon in Asia, PDB has been increasingly reported from the Indian subcontinent over the last two decades though the data are primarily in the form of case reports / case series [9, 20–22]. In a series of 28,000 patients with diabetes, Paget’s disease was estimated in 0.066% [23].

There are reports of patients with Paget’s disease of Asian (Indian subcontinent) origin living in the UK and New Zealand though the numbers of patients is small [14, 24]. The reason for the diagnosed low prevalence among those identifying as Asians and Asian British in Greater Manchester, compared to whites or those who identify as Black or Black British is unknown. Both genetic and environmental factors are involved in the pathogenesis of Paget’s, and it is possible variation may be due to differences in one or more susceptibility factors and for which further research is needed.

In our data Paget’s disease was associated with a small though non-significant increased risk of severe COVID. Not everyone, however, who tested positive for COVID was recorded on the dataset and not all admissions during this time were due to COVID. Any misclassification due to underreporting and to non-COVID related admissions seems unlikely to be related to the occurrence of disease and would probably tend to reduce the likelihood of finding a biologic association. We were not able to adjust the findings for comorbid factors, which may have been linked with Paget’s and also adverse COVID outcome. There was evidence also of an increased risk of severe COVID (admission to hospital) linked with increasing age, gender (men > women) and increasing levels of deprivation [25].

Our data is based on population sample of people registered with their GP. There are important limitations to be considered when interpreting our findings. Classification of ethnicity was based on self-report and many people declined to define their ethnicity while others classified themselves as mixed race; the latter included those of white and Caribbean background, white and black African and white and Asian and other mixed race.

Misclassification of ethnic status may have potentially resulted in either an under- or over estimation of the true occurrence of disease among individual ethnic groups. Any such misclassification is, however, if anything to reduce the likelihood of finding significant biologic associations. As outlined our data relate to those with clinically diagnosed disease. Factors influencing clinical presentation, including for example comorbidity and health seeking behaviour may potentially impact on the likelihood of an individual being diagnosed with the disease and it is possible that such factors may explain some of the observed variation in occurrence by ethnic group. Our data concerning deprivation is derived from census data and based on current residence; and may not therefore reflect levels of deprivation experienced during the life course. Finally, our data are based on data from a large urban conurbation in the northwest of England and some caution is needed in extrapolating the findings beyond this group.

In summary, the prevalence of clinically diagnosed Paget’s disease of bone in Greater Manchester in 2020 was 0.174%. Prevalence increased with increasing deprivation and was compared to whites, more common among those who identified as Black or Black British and less common among those who identified as Asian or Asian British. Further research is required to confirm these findings and to determine whether such differences are due to variation in disease occurrence or disease presentation and also the causes of such variation.
